# The impact of racial discrimination on the health of Australian Indigenous children aged 5–10 years: analysis of national longitudinal data

**DOI:** 10.1186/s12939-017-0612-0

**Published:** 2017-07-03

**Authors:** Carrington C.J. Shepherd, Jianghong Li, Matthew N. Cooper, Katrina D. Hopkins, Brad M. Farrant

**Affiliations:** 1 Telethon Kids Institute, The University of Western Australia, P.O. Box 855, West Perth, WA 6872 Australia; 2 WZB Berlin Social Science Center, Reichpietschufer 50, 10785 Berlin, Germany; 3 Centre for Population Health Research, Curtin University, Kent Street, Bentley, WA 6102 Australia

**Keywords:** Racism, Aboriginal, Indigenous, Mental health, Physical health, Australia

## Abstract

**Background:**

A growing body of literature highlights that racial discrimination has negative impacts on child health, although most studies have been limited to an examination of direct forms of racism using cross-sectional data. We aim to provide further insights on the impact of early exposure to racism on child health using longitudinal data among Indigenous children in Australia and multiple indicators of racial discrimination.

**Methods:**

We used data on 1239 Indigenous children aged 5–10 years from Waves 1–6 (2008–2013) of *Footprints in Time*, a longitudinal study of Indigenous children across Australia. We examined associations between three dimensions of carer-reported racial discrimination (measuring the direct experiences of children and vicarious exposure by their primary carer and family) and a range of physical and mental health outcomes. Analysis was conducted using multivariate logistic regression within a multilevel framework.

**Results:**

Two-fifths (40%) of primary carers, 45% of families and 14% of Indigenous children aged 5–10 years were reported to have experienced racial discrimination at some point in time, with 28–40% of these experiencing it persistently (reported at multiple time points). Primary carer and child experiences of racial discrimination were each associated with poor child mental health status (high risk of clinically significant emotional or behavioural difficulties), sleep difficulties, obesity and asthma, but not with child general health or injury. Children exposed to persistent vicarious racial discrimination were more likely to have sleep difficulties and asthma in multivariate models than those with a time-limited exposure.

**Conclusions:**

The findings indicate that direct and persistent vicarious racial discrimination are detrimental to the physical and mental health of Indigenous children in Australia, and suggest that prolonged and more frequent exposure to racial discrimination that starts in the early lifecourse can impact on multiple domains of health in later life. Tackling and reducing racism should be an integral part of policy and intervention aimed at improving the health of Australian Indigenous children and thereby reducing health disparities between Indigenous and non-Indigenous children.

**Electronic supplementary material:**

The online version of this article (doi:10.1186/s12939-017-0612-0) contains supplementary material, which is available to authorized users.

## Background

Conventional frameworks of population health typically implicate a range of social, environmental and economic factors as key determinants of health [1]. However, there are historical and contemporary factors such as dispossession, discrimination and racism, that are uniquely experienced by minority and Indigenous populations and are known to impact on mental and physical health [2–4]. Racism, in particular, is being cited more commonly in the international literature as having serious adverse consequences for health [5–10], and is widely acknowledged as playing a crucial role in the formation of health disparities [11–14].

Racism takes many forms and can be expressed at interpersonal, systemic and internalised levels [15–18]. Accordingly, the pathways by which racism impacts on health are multifaceted and complex, for all populations [7, 19]. They include the direct impacts of racism on health (e.g. racially motivated physical assault), and the indirect effects that stem from reduced and unequal access to medical, social and economic resources required for good health, increased exposure to risk factors associated with ill health, such as withdrawal from health care and health promoting activities, and the stress of racism and its ill effects [20, 21]. In addition, poor health in childhood has been linked to the experience of vicarious racism—that is, the experiences of others, including parents, carers and family members [22]. Few studies have examined how vicarious racism impacts on child health and wellbeing, although the hypothesised mechanisms are centred around the stress associated with parental experiences of (direct and vicarious) racism and the resultant impact on parenting practices [23–25].

Despite a considerable increase in the number of studies on racism and health in recent years, relatively few have focused on younger age groups [5, 26, 27]. The existing research has primarily examined the impacts of direct forms of racism, and suggests that younger people are more vulnerable to racism’s harmful effects. These effects extend to negative outcomes in general health, life satisfaction, behaviour problems, health-related behaviours (alcohol, drug use and smoking), and healthcare utilization [22]. In addition, a small number of studies have shown that parent-experienced racial discrimination is associated with pregnancy and birth outcomes [28–32]. While this signifies that racism can influence development in the very early stages of life, few studies have examined the ongoing consequences of early exposure to this form of stress [33]. The sparse evidence from studies that draw on longitudinal data indicates that racism precedes poor health but provides few insights about the sensitivity of early exposure, the importance of duration of exposure, or the latency period between exposure to racism and onset of illness; as a result, the causal pathways through childhood and later life are not fully understood [5, 33].

Most studies of the impact of racial discrimination in childhood have been conducted in the United States among African American, Latino and Asian groups, with few studies in other minority populations and national contexts [22]. The quantitative empirical evidence for Indigenous populations is particularly sparse, despite the fact that racism is commonly cited as underpinning Indigenous people’s experiences of exclusion from aspects of social, political, and economic life [8, 34]. In Australia, for example, there have only been a handful of studies on populations of Indigenous (Aboriginal and Torres Strait Islander) children and these have focused on a restricted set of mental health, health risk behaviour and physical health indicators using primarily cross-sectional data for discrete population groups [35–38]. While the results in Australian Indigenous settings are broadly consistent with the US literature [22], much more research is needed in order to understand the role of racism as a determinant of Indigenous child health and the underpinning pathways.

The objective of this study is to use national longitudinal data on Indigenous children and multiple-indicators of racial discrimination to provide further insights on the links between persistent racism and health in early life. We use three dimensions of carer-reported racial discrimination that capture the direct experiences of children themselves and also vicarious exposure by their primary carer and family. Six waves of data from the *Footprints in Time* study (the Longitudinal Study of Indigenous Children; hereafter referred to as LSIC) are drawn upon to examine the associations between racial discrimination and a range of health outcomes that have been shown to be associated with racism in prior research on ethnic population groups. These outcomes include mental health [39], general health [37], sleep difficulties [40, 41] and obesity [42]. In addition, we examine injury as an outcome given the direct threats to health posed by racial violence and the indirect pathways from racism-related stress to mental impairment and unintentional injuries [7], and asthma, to further test the relationship found between psychosocial stress and the risk to poor child respiratory health [43]. In addition, we use population attributable risks (PARs) to further assess the significance of racism as a determinant of Australian Indigenous child health. PAR estimates consider both the level of exposure to a risk factor and its impact on a health outcome, and provide a measure of how much disease can be averted if the risk factor could be eliminated [44].

## Methods

The LSIC is the first large-scale longitudinal survey to focus on the development of Australian Indigenous children [45]. The first wave of the survey was conducted in 2008, with subsequent waves conducted annually. The LSIC employed a non-random purposive sampling design, with study children recruited from 11 sites across Australia. These sites were chosen for practical and logistical reasons, and to represent the diversity of community environments where Indigenous children live [46]. Further details regarding the rationale, sampling, recruitment, and data collection have been reported elsewhere [47].

### Participants

The participants for the current study included 1239 Indigenous children retained in the Wave 6 sample of the LSIC. The LSIC originally recruited two cohorts of Indigenous children in 2008 using purposive sampling. Families were approached within study sites based on address records provided by Centrelink and Medicare Australia of potentially in-scope children. Word of mouth and study promotion were also employed to recruit additional children. LSIC study children included individuals identified by their (consenting) primary carer as Indigenous and (1) aged between 6 months and 2 years at Wave 1 (born between 2006 and 2008; *n* = 954), or (2) aged between 3½ and 5 years at Wave 1 (born between 2003 and 2005; *n* = 717). By Wave 6 in 2013, the two cohorts had reached 5½–7 and 8½–10 years of age, respectively. A total of 1671 children were recruited for Wave 1, and these children were involved in subsequent waves where possible. The sample retention was 74% at Wave 6. At Wave 1, 94.9% of the children spoke English, 20.0% spoke an Indigenous language, and 15.0% spoke both English and an Indigenous language [48]. The majority (86.8%) of the children were identified by the parent as Aboriginal with 7.2% Torres Strait Islander and 6.0% identified as being both.

The primary respondent at each wave was the child’s primary parent or carer. The carer who completed the Wave 1 interview was the birth mother in 93.9% of cases and the data reported here relate to responses from the person identified as the primary carer.

### Primary explanatory variables

The LSIC asked a suite of questions on issues of racism, discrimination and prejudice. We included three questions in this study: the primary carer’s views on their own experiences of racism (“Have you been treated unfairly or discriminated against or been treated badly because you are Aboriginal/Torres Strait Islander?”), and their perceptions of the experiences of their family (“How often does your family experience racism, discrimination or prejudice?”) and the study child at school (“As far as you know, has the study child been bullied or treated unfairly at preschool/school because he/she is Aboriginal or Torres Strait Islander?”). These three responses provide both indirect (vicarious) and direct measures of the study child’s exposure to interpersonal (and potentially institutionalised) racism. For analysis purposes, persistent racial discrimination was defined in this study as exposure in multiple waves. Data for primary carer exposure was collected in Waves 1 and 4 and in Waves 3 and 5 for family exposure; while child exposure was collected in Waves 2 and 6 (older cohort) and Waves 4 and 5 (younger cohort), small cell sizes precluded a robust statistical analysis of persistent exposure to racism at school among study children. Exposure in a single wave only was categorised as time-limited racial discrimination. Only valid responses at both time points were used to define persistent and time-limited exposure.

### Outcome measures

Overall, six indicators were selected for analysis in this study—one mental health and five physical health measures. Mental health (or social and emotional wellbeing) was measured using the Strengths and Difficulties Questionnaire (SDQ) in Wave 6. The SDQ assessed risk status for clinically significant emotional or behavioural difficulties (CSEBD) [49, 50]. Consistent with its design parameters, the SDQ was collected only for participants aged 3 years and over. The 20 questions that examined emotional symptoms, conduct problems, hyperactivity and peer problems were combined to produce a SDQ Total Score (range 0–40). Primary carers’ responses to the SDQ form the basis of the analysis of Indigenous children’s mental health in this study, with scores of 17–40 indicating that a child was at high risk of CSEBD (or a mental health problem).

The physical health indicators were primarily chosen on the basis of prior research on the relationship between racism and physical health among ethnic population groups [5, 12, 22]. Primary carers were asked in Wave 6 about the study child’s general health, whether they experienced sleep difficulties, weight status, and whether the child had ever had an injury or asthma: (1) the subjective measure of general health was assessed by carers using a five-point scale. Responses were dichotomised, with children recoded as being in either ‘excellent/very good’ health or ‘poor, fair or good’ health; (2) children who usually had trouble getting to sleep or staying asleep in the last month were deemed to have sleep difficulties, based on responses from the latest available wave (Wave 6 for the younger cohort and Wave 5 for the older cohort); (3) weight status was derived from height and weight measurements taken by LSIC interviewers. Children were categorised as underweight, normal weight, overweight, or obese based on body mass index (BMI) z-scores [51]. Obesity was the outcome of interest in this study, with comparisons focusing on children categorised as ‘obese’ vs. ‘normal weight’; (4) injuries included fractures, dislocations, sprains, burns, cuts, concussion, internal injuries, dental injuries, unintentional poisoning, near drowning, dog bites and other injuries. Children that had experienced at least one of these in their life were coded as having had an injury for the purposes of this study; and (5) to determine asthma prevalence, carers were simply asked whether the child had ‘ever had asthma’.

### Covariates

A number of variables that may also influence the study outcomes were considered as covariates in the analyses. These include demographic characteristics (child sex, the age of the study child and primary carer, and level of relative geographic isolation) and socioeconomic status (SES) indicators (primary carer education, material wellbeing and measures of neighbourhood/community SES). Geographic isolation is defined using the Level of Relative Isolation (LORI) classification, which is based on the Accessibility/Remoteness Index of Australia (a widely used classification of remoteness in Australia). The separate categories of isolation reflect differences in access to services, cultures and health outcomes for Indigenous children in Australia [52]. The LORI of each study household was recorded at each wave on a four-point scale that ranged from none (metropolitan area), to low, moderate and high/extreme. The most recently recorded location was used in the present analyses. Primary carer education was reported at Waves 2 and 3 on a fourteen-point scale that ranged from never attended school to postgraduate degree. In the present study the most recently reported value was used. We tested two constructs of material wellbeing—financial strain and family income. The former was considered the superior measure for this study, on both practical and theoretical grounds, as (1) the odds ratios for family income were generally close to the null in models testing the association between health and racism, and (2) income does not capture the nature of sharing of economic resources that can occur between members of extended Indigenous families [53]. The subjective rating of financial strain was coded to five categories: from “we can save a lot” to “we run out of money before payday”. Two measures of neighbourhood/community SES were tested—the Australian Bureau of Statistics’ Socioeconomic Index for Areas (SEIFA) product and Biddle’s Index of Relative Indigenous Socioeconomic Outcomes (IRISEO). Both SEIFA and IRISEO were generally found to be unrelated to aspects of Indigenous child wellbeing in this study and were removed from the final models presented in this paper.

### Analysis

The analyses undertaken for this study is based on the information available for 1239 children at Wave 6 of the LSIC. In preliminary analyses, we calculated the prevalence of the three racial discrimination variables, with logistic regression models used to quantify the independent associations between these variables and the socio-demographic covariates described above (all variables were adjusted for simultaneously). The primary analyses—examining the associations between racial discrimination and health at Wave 6 (5–10 years of age)—were also conducted using logistic regression. All physical and mental health outcome measures were dichotomised—total SDQ score, general health, sleep difficulties, weight status (obesity), whether suffered an injury and asthma—and modelled separately with each racism variable. Two models were constructed to examine the relationship between each racism and health combination. The first (Model 1) includes the age and sex of the study child as basic covariates. In the second model (Model 2) we also included the age of the primary carer, level of relative geographic isolation of the household, primary carer education and family financial strain, and tested whether their inclusion varies the strength or nature of the association between racism and health. We examined the change in effect size between Models 1 and 2 as an indication of the robustness of the relationship between racism and health. A multilevel approach was employed in all modelling to account for both the survey’s clustered sampling design and the repeated measures on the same individual [54]. SAS version 9.4 (SAS Institute Inc., Cary, NC, USA, 2002–12) was used for all analyses.

Population attributable risks (PARs) were derived for each racial discrimination variable using the adjusted odds ratios (aOR) from Model 2. We report odds ratios (OR) and PARs, with 95% confidence intervals (95% CI). Confidence intervals (95%) around PAR values were calculated using the method described by Hildebrandt et al. (2006) [55].

Primary exposures, outcomes and covariates were subject to non-response, with regression models based on participants with complete data. The models used in preliminary analyses included complete data for most of the sample at Wave 6 (1020–1196 cases); in the primary analyses, the models for primary carer (561–791 cases) and family (684–961 cases) experiences of racial discrimination had fewer complete cases—this was mostly the result of not having valid data in multiple waves in order to define persistent and time-limited exposure to racial discrimination. To assess the impact of missing data on the effect estimates, a sensitivity analysis was carried out using imputed data. Multivariate imputation by chained equations was used to generate 10 data sets [56], the same models were run and the coefficients were combined and compared to the coefficients generated using complete cases only. These analyses were conducted using R (version 3) [57].

## Results

Almost a quarter (23%) of Indigenous children in Wave 6 of the LSIC had a mental health problem (high risk of clinically significant emotional or behavioural difficulties). In addition, 25% had their health assessed as less than excellent or very good, 21% had difficulty sleeping in the last month, 21% were obese, 4% had suffered an injury in their lifetime, and 11% had asthma.

**Fig. 1:**
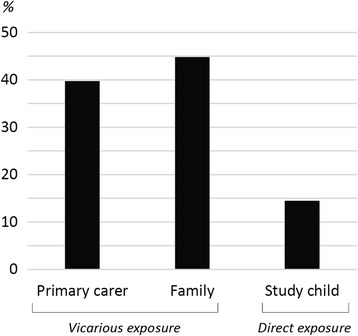
Proportion of Indigenous children aged 5–10 years that have ever been exposed to racial discrimination

Two-fifths (40%) of primary carers, 45% of families and 14% of Indigenous children aged 5–10 years were reported to have experienced racial discrimination at some point in time (Fig. 1). The majority (69%) of primary carers that had experienced racial discrimination had a time-limited exposure (reported in one wave only), while 31% experienced persistent racial discrimination (reported in multiple waves). A similar pattern was observed for family members (60% time-limited and 40% persistent) and study children (72% and 28%, respectively).

Table 1 includes odds ratios from logistic regression analyses between exposure to racism and socio-demographic factors, and highlights that there were generally only weak to moderate independent associations observed, with few statistically significant effects. The results, however, indicate that it was more common for older Indigenous children and primary carers to have experienced racial discrimination. In particular, children aged 9–10 years were over 4 times more likely (OR = 4.4; 95% CI: 2.85–6.79) to be treated badly or discriminated against because they were Indigenous than children aged 5–6 years. Racial discrimination appeared to be associated with geographic location: primary carers and families living in areas of moderate and high/extreme isolation were less likely to ever be exposed to racial discrimination, with those living in the most remote locations having the lowest odds (OR = 0.43, 95% CI: 0.24–0.78 for primary carers, and OR = 0.22, 95% CI: 0.12–0.40 for families) relative to children in metropolitan areas. In contrast, children in high/extreme isolation had elevated odds of exposure to racism at school (OR = 2.2; 95% CI: 1.08–4.44). The results for SES were mixed: the evidence for a relationship between reported racial discrimination and the educational attainment of the primary carer was inconclusive in that only a relatively weak and statistically insignificant odds ratio for primary carer and family experiences of racism was observed among those with a bachelor degree or higher. However, there was a stronger and statistically significant association for family financial strain, with an increasing likelihood of exposure to racial discrimination for children in families with poorer financial circumstances.

**Table 1:** Multivariate association between experiences of racial discrimination and selected socio-demographic characteristics of Indigenous children

		Primary carer’s experience of racism (ever)		Family experience of racism, discrimination or prejudice (ever)		Study child treated badly or discriminated against because they were Indigenous (ever)	
	% (n)^a^	aOR	95% CI	aOR	95% CI	aOR	95% CI
Sex of study child							
Male	50.0 (628)	Reference	..	Reference	..	Reference	..
Female	50.0 (627)	0.95	0.73–1.24	0.98	0.78–1.25	1.19	0.82–1.72
Age of study child (years)							
5–6	50.0 (628)	Reference	..	Reference	..	Reference	..
7–8	12.4 (156)	0.91	0.60–1.38	0.82	0.56–1.20	1.61	0.84–3.11
9–10	37.5 (471)	1.04	0.78–1.39	0.86	0.66–1.12	4.40**	2.85–6.79
Age of primary carer (years)							
20–29	25.2 (316)	Reference	..	Reference	..	Reference	..
30–39	46.8 (588)	1.20	0.86–1.69	1.14	0.84–1.55	0.88	0.54–1.44
40–49	21.3 (267)	1.74**	1.17–2.59	1.69**	1.17–2.43	0.95	0.54–1.67
50 and over	6.7 (84)	1.61*	0.92–2.81	1.34	0.79–2.28	0.77	0.34–1.77
Level of relative isolation							
None	27.6 (347)	Reference	..	Reference	..	Reference	..
Low	50.4 (633)	1.05	0.74–1.51	0.94	0.70–1.26	0.83	0.50–1.37
Moderate	12.9 (162)	0.68	0.41–1.12	0.47**	0.30–0.73	1.49	0.75–2.95
High/extreme	9.0 (113)	0.43**	0.24–0.78	0.22**	0.12–0.40	2.19**	1.08–4.44
Education: primary carer							
Bachelor degree or above	8.8 (109)	1.40	0.82–2.39	1.41	0.88–2.26	1.19	0.57–2.51
Other post-school	39.3 (484)	0.97	0.71–1.34	1.31*	0.97–1.76	1.19	0.75–1.89
Year 11 or 12	22.9 (282)	0.86	0.59–1.25	0.77	0.54–1.09	1.25	0.74–2.12
Year 10 or below	29.0 (357)	Reference	..	Reference	..	Reference	..
Family financial strain							
Save a lot	6.2 (78)	Reference	..	Reference	..	Reference	..
Save a bit every now and then	39.9 (501)	1.33	0.71–2.47	0.99	0.59–1.70	0.78	0.37–1.67
Some money left over each week but we just spend it	9.2 (116)	1.28	0.60–2.69	1.18	0.63–2.21	0.49	0.18–1.33
Just enough money to get us through to the next pay	33.3 (418)	1.88*	1.00–3.54	1.41	0.82–2.40	1.10	0.51–2.39
Spending more than we get	11.2 (141)	2.20**	1.08–4.48	1.23	0.67–2.24	0.85	0.34–2.12

*aOR* adjusted odds ratio, ** *p* value <0.05; * *p* value <0.10 (marginal statistical significance)

Note: The table includes separate models for: primary carer’s experience of racism (*n* = 1020); family experience of racism, discrimination or prejudice (*n* = 1196); and study child treated badly or discriminated against because they were Indigenous (*n* = 1109). Models exclude records with missing data. Each model is adjusted for sex and age of the study child, age of the primary carer, level of relative isolation, primary carer education, and family financial strain

^a^Full Wave 6 sample

Table 2 highlights a generally positive association between primary carer and child experiences of racial discrimination and poor physical and mental health for Indigenous children. This includes statistically significant effects (*p* < 0.05) for mental health, sleep difficulties and asthma (with the ORs ranging from about 1.7 to 2.7). While some of the associations for obesity reached marginal significance (*p* < 0.10), there was no discernible association with general health and injury. In contrast, there appeared to be only weak and non-significant associations between the selected health indicators and family experiences of racial discrimination. Modelling using multiple imputation was carried out for both mental health (no missing outcome data, Additional file 1: Table S1) and obesity (some missing outcome data, Additional file 2: Table S2). Effect size estimates were consistent (differed only at the second decimal place) with analyses conducted on complete cases only so this method was not pursued.

**Table 2:** Odds ratios and PAR proportions for associations between health outcomes and experiences of racial discrimination

Experiences of racial discrimination	Model 1^a^		Model 2^b^			
	OR	95% CI	aOR	95% CI	PAR (%)	95% CI
*Mental health*						
Primary carer^c^						
No exposure	Ref.	Ref.	Ref.	Ref.	..	..
Time-limited (one wave)	0.84	0.56–0.26	0.78	0.51–1.19	−6.8	−14.5, 1.0
Persistent (two waves)	1.68**	1.02–2.74	1.46	0.88–2.45	5.8	0.3, 11.2
Family^c^						
No exposure	Ref.	Ref.	Ref.	Ref.	..	..
Time-limited (one wave)	1.00	0.69–1.45	0.95	0.64–1.39	−1.4	−9.0, 6.1
Persistent (two waves)	1.16	0.76–1.78	1.12	0.71–1.76	2.2	−3.7, 8.0
Child						
No exposure	Ref.	Ref.	Ref.	Ref.	..	..
Ever exposed	2.21**	1.48–3.30	2.32**	1.52–3.53	16.2	11.1, 21.2
*General health*						
Primary carer^c^						
No exposure	Ref.	Ref.	Ref.	Ref.	..	..
Time-limited (one wave)	1.11	0.75–1.64	1.14	0.76–1.70	3.9	−3.8, 11.6
Persistent (two waves)	0.79	0.45–1.38	0.78	0.44–1.38	−3.0	−7.4, 1.4
Family^c^						
No exposure	Ref.	Ref.	Ref.	Ref.	..	..
Time-limited (one wave)	0.99	0.69–1.43	1.05	0.73–1.52	1.4	−5.9, 8.7
Persistent (two waves)	0.64*	0.40–1.03	0.70	0.43–1.13	−5.9	−10.0, −0.9
Child						
No exposure	Ref.	Ref.	Ref.	Ref.	..	..
Ever exposed	1.34	0.89–2.03	1.31	0.86–1.98	4.3	−0.3, 8.9
*Sleep difficulties*						
Primary carer^c^						
No exposure	Ref.	Ref.	Ref.	Ref.	..	..
Time-limited (one wave)	0.73	0.47–1.12	0.69	0.44–1.08	−9.6	−17.6, −1.6
Persistent (two waves)	2.16**	1.34–3.48	1.92**	1.17–3.15	10.8	4.6, 17.0
Family^c^						
No exposure	Ref.	Ref.	Ref.	Ref.	..	..
Time-limited (one wave)	1.21	0.84–1.75	1.10	0.76–1.59	2.8	−5.0, 10.5
Persistent (two waves)	1.43*	0.95–2.16	1.17	0.77–1.79	3.1	−3.0, 9.2
Child						
No exposure	Ref.	Ref.	Ref.	Ref.	..	..
Ever exposed	2.36**	1.54–3.61	2.72**	1.77–4.18	19.1	13.8, 24.4
*Obesity*						
Primary carer^c^						
No exposure	Ref.	Ref.	Ref.	Ref.	..	..
Time-limited (one wave)	1.44	0.89–2.31	1.50	0.92–2.44	13.1	2.2, 23.9
Persistent (two waves)	1.52	0.80–2.87	1.54	0.80–2.97	6.3	−0.2, 12.9
Family^c^						
No exposure	Ref.	Ref.	Ref.	Ref.	..	..
Time-limited (one wave)	1.26	0.83–1.92	1.24	0.81–1.90	6.6	−2.4, 15.7
Persistent (two waves)	1.13	0.68–1.89	1.03	0.61–1.73	0.5	−5.9, 6.9
Child						
No exposure	Ref.	Ref.	Ref.	Ref.	..	..
Ever exposed	1.41	0.86–2.32	1.63*	0.98–2.70	8.2	2.2, 14.1
*Injury*						
Primary carer^c^						
No exposure	Ref.	Ref.	Ref.	Ref.	..	..
Time-limited (one wave)	0.79	0.31–2.04	0.79	0.29–2.14	−6.4	−25.7, 12.9
Persistent (two waves)	1.84	0.67–5.00	1.52	0.53–4.37	6.5	−9.2, 22.1
Family^c^						
No exposure	Ref.	Ref.	Ref.	Ref.	..	..
Time-limited (one wave)	0.52	0.20–1.36	0.50	0.19–1.31	−16.7	−34.5, 1.1
Persistent (two waves)	0.88	0.35–2.23	0.75	0.28–1.97	−4.9	−21.3, 11.5
Child						
No exposure	Ref.	Ref.	Ref.	Ref.	..	..
Ever exposed	1.07	0.46–2.50	0.88	0.35–2.21	−1.8	−14.5, 11.0
*Asthma*						
Primary carer^c^						
No exposure	Ref.	Ref.	Ref.	Ref.	..	..
Time-limited (one wave)	1.24	0.73–2.11	1.28	0.74–2.21	7.5	−4.6, 19.5
Persistent (two waves)	2.65**	1.47–4.76	2.56**	1.40–4.69	17.2	7.8, 26.5
Family^c^						
No exposure	Ref.	Ref.	Ref.	Ref.	..	..
Time-limited (one wave)	1.30	0.80–2.10	1.24	0.76–2.03	6.4	−5.3, 18.1
Persistent (two waves)	1.32	0.76–2.30	1.17	0.66–2.07	3.1	−5.9, 12.0
Child						
No exposure	Ref.	Ref.	Ref.	Ref.	..	..
Ever exposed	1.77**	1.05–3.00	2.04**	1.19–3.48	13.2	5.6, 20.7

*aOR* adjusted odds ratio, ** *p* value <0.05; * *p* value <0.10 (marginal statistical significance); PAR = population attributable risk

Note: The table includes separate models for each of the three racism variables by six indicators of health. Models exclude records with missing data

^a^Model 1 is adjusted for sex and age of the study child

^b^Model 2 includes Model 1 variables as well as the age of the primary carer, level of relative isolation, primary carer education, and family financial strain

^c^‘Time-limited’ is defined as exposure to racism in one wave only, while ‘Persistent’ includes exposure in multiple waves

The effect sizes for the associations between primary carer experiences of racial discrimination and child health tended to be considerably larger for those exposed to persistent racial discrimination when compared with those who experienced time-limited racial discrimination, suggesting a dose-response relationship between this form of vicarious racism and mental health, sleep difficulties and asthma. For example, children were 2.6 times more likely (95% CI: 1.47–4.76) to have asthma when their primary carer experienced persistent racial discrimination, with a weak and non-significant odds (OR = 1.2, 95% CI: 0.73–2.11) when the carer reported racial discrimination in one wave only.

The relationships between racial discrimination and health are only slightly attenuated by the inclusion of other known covariates in the statistical models. This suggests that the socio-demographic circumstances of Indigenous families have little impact on the relationship between racism exposure and the health of children.

Adjusting for all variables included in Model 2, we found the highest PARs for child experiences of racism. This accounted for 16.2% (95% CI: 11.1–21.2%) of the PAR for mental health status, 19.1% (95% CI: 13.8–24.4%) of the PAR for sleep difficulties, 8.2% (95% CI: 2.2–14.1%) of the PAR for obesity and 13.2% (95% CI: 5.6–20.7%) of the PAR for asthma. Carer experiences of persistent racial discrimination accounted for a portion of the PAR for mental health (5.8%; 95% CI: 0.3–11.2%), sleep difficulties (10.8%; 95% CI: 4.6–17.0%) and asthma (17.2%; 95% CI: 7.8–26.5%), while time-limited exposure accounted for 13.1% (95% CI: 2.2–23.9%) of the PAR for obesity.

## Discussion

This study is one of the first to use longitudinal data to examine the effects of both direct and vicarious racial discrimination on a range of child health indicators. We have shown that racism is associated with mental health and aspects of the physical health of Indigenous children in Australia, with the most consistent results relating to mental health status, sleep difficulties, obesity and asthma. While prior studies suggest that racism poses the greatest risk to mental health [22], children experiencing racial discrimination in this study were, broadly speaking, at an equally elevated risk of having sleep difficulties, asthma and mental health problems.

In a novel finding, we highlight the negative impact that persistent vicarious racism has on child health, with reports of racial discrimination by primary carers at two earlier time points associated with a 1.7–2.6 times increased odds of poor mental health, sleep difficulties and asthma in children at age 5–10 years. This finding is consistent with a causal association between racism and health, and suggests that prolonged and/or more frequent exposure to vicarious racial discrimination that starts in the early lifecourse can impact on multiple domains of health in later life. This is likely to reflect processes of biological embedding, whereby frequent exposure to stressors in early life can impact on the development and functioning of vital organs, and contribute to systematic differences in health throughout life [58, 59]. These pathways are, plausibly, enmeshed with the experiences of racism among parents and the effect that this has on their mental wellbeing and ability to provide support that optimises child development [24, 25]. While relatively little is known about the pathways from vicarious racism to poor child health, an increased focus on longitudinal studies that begin at birth will provide a deeper understanding of the role of this form of racism in the pathways to disadvantage, including the persistent and striking inequalities in health between Indigenous and other populations.

The magnitude of elevated risk for mental health found in our study is consistent with existing studies in Australian Indigenous settings, and suggests that the direct experience of racial discrimination (assessed using single-item measures) is associated with a doubling of the risk of an Indigenous child or youth developing a mental health problem [36–38]. This includes a diverse set of mental health outcomes, such as anxiety, depression, suicide risk, low self-esteem, as well as overall mental health status. Priest et al. (2011) showed a higher risk among Indigenous youths (odds ratio of 3.35) which may reflect the older ages of study participants in the study (16–20 year-olds) or the fact that participants reported their own experiences of racism and mental health [38]. Similar studies among other minority groups also predominantly focus on adolescents and youth; one study of fifth-grade students (usually aged 10–11 years) in US metropolitan areas highlighted that children from non-White racial/ethnic groups who perceived discrimination were up to 3.9 times more likely to suffer depression and up to 2.4 times more likely to have specific externalizing disorders, with differences in relative risk between Black, Hispanic and other ethnic groups [60]. These results and the findings from our study confirm that, while children may be less likely to perceive discrimination in the period prior to adolescence, it nonetheless poses a considerable risk to the mental wellbeing of those who perceive it.

Our study highlights a relationship between racism and sleep difficulties, although the strength of this association appears to be greater than the results of previous studies in the US and Brazil [40, 41]. The findings of Bittencourt et al. (2009) and Huynh and Gillen-O’Neel (2013) suggest that both overt and subtle forms of discrimination have a moderate impact on the amount and quality of sleep among adolescents from ethnic minority populations. Our study supports the notion that stress associated with racism can also disrupt sleep in children, although further research is needed to quantify the impacts on quality and duration of sleep, particularly given the importance of sleep to brain development in childhood [61] and ongoing wellbeing [62].

Previous studies have also highlighted that parental stress exacerbates the causal risks of childhood asthma [43], although to our knowledge no studies have assessed racism specifically as a stressor. We have shown that both parental and children’s direct experiences of racism are strongly associated with asthma, with a 2–3 times greater risk for these racism exposures in multivariate models. Racism is likely to impact asthma in multiple ways—for example, the stressful effects of racism can impact on multiple bodily systems and increase a person’s susceptibility to the range of environmental exposures that are implicated in the development of asthma (e.g. air pollution and smoke exposure). In addition, the association may partly be an artefact of greater visibility of asthma responses in those experiencing racism, reflecting the connection between stress events and the onset of wheeze and asthma attacks in children [63, 64].

The pervasive health inequalities between Indigenous and non-Indigenous people in Australia demands a better understanding of the aetiology of poor health outcomes in Indigenous populations. While the current scientific literature implicates racism and its ill effects in the complex pathways to physical and mental health problems [5], there has been little scrutiny of the saliency of these factors in Indigenous children. This study adds to the sparse studies on Indigenous children, and further confirms that Indigenous children exposed to racism tend to be at a considerably greater risk of poor health [35–38]. In addition, the prevalence of racism among participants in this study is broadly consistent with the existing estimates in comparable studies of Indigenous child populations. We found that 40% of primary carers, 45% of families and 14% of Indigenous children aged 5–10 years had experienced racism at some point in time, with previous studies using single-item measures typically in the range of 15–40% [22]. However, the prevalence of direct experiences of racism among 12–17 year olds in Victoria (35%) and Western Australia (22%) are higher than what we have reported in this study (14%). This is likely to reflect differences in participant age range and respondent awareness. Prevalence rates are generally higher among adolescents than children, reflecting that adolescence is a time when children widen their spheres of influence and gain a greater awareness of discrimination [25]. Further, previous studies have asked adolescents about their experiences of racism directly whereas the LSIC asked the primary carer about their (perhaps limited) perceptions of their child’s experiences.

PAR estimates were of a low to moderate size, with notable values shown for mental health, sleep difficulties, obesity and asthma with respect to both child and primary carer experiences of racial discrimination. PARs can highlight modifiable risk factors and estimate how much disease can be averted with their elimination [44], although few studies have examined the PAR of racism, to date. The results here suggest that eradicating racism is likely to reduce the burden associated with some important health problems in childhood and therefore account for a portion of the health inequality between Indigenous and other Australians. The PAR estimates here do not fully capture the impact of racism on health inequality, given that the effect of racism on other determinants of health was not examined in this study due to limitations in the available data.

Overall, the study results suggest that children who are exposed to racial discrimination (and/or perceive it) have poorer physical and mental health, and that racism is an important modifiable risk factor that needs to be targeted to reduce disease burden. However, the impact of racism needs to be explored in more detail in order to more fully understand what types of racism have the most impact on Indigenous health. We need to know more about the prevalence of systemic, interpersonal and internalised racism for Indigenous Australians and how this varies by place and socioeconomic status, among other factors. Instruments need to be developed that measure perceptions of racism in children and youth that take into account the unique contexts and developmental levels of children. And, critically, future research will need to focus on pathways in order to deepen our understanding of detrimental effects of racism on health. All of this will help us design more effective interventions to combat racism. This, in turn, will help us to increase the pace of change toward health equality in Australia.

### Strengths and limitations

The main strengths of this study are that it: (1) draws upon a large, national dataset that was collected using robust and culturally appropriate methods and processes; (2) utilizes validated and reliable tools for assessing social and emotional wellbeing (mental health); (3) employs rigorous analytical methods; (4) uses a wide range of indicators that measure different aspects of racism and health in the Australian Indigenous population, including measures of racism at different points in time; (5) reduces the likelihood of recall bias given multiple surveying of participants; and (6) reports two measures of the effect of racism, including PARs which can support a better understanding of the importance of racism as a health risk factor.

The limitations mainly relate to the measurement of the construct of racism. First, while three dimensions of racial discrimination were presented here, they only reflect the perceptions of the primary carer, which may not necessarily accord with the views and experiences of study children or other family members. Second, racism is a complex, multifaceted concept that goes beyond the scope of interpersonal racism, which was the primary focus of our analysis. Third, we used only single-item measures of racism which typically lead to an under-estimation of the extent of racism experience [16]. Fourth, there are likely to be a diversity of responses to situations of racial prejudice and discrimination among study respondents. Responses can be influenced by many factors—including those related to identity, behaviour, capabilities, self-esteem, social support, and peer and inter-ethnic relations, among others—and may therefore shape the recording of racism prevalence and its relationship with child health. In addition, the LSIC employed a non-random sampling design, with study sites chosen for practical and logistical reasons. While these sites represent the diversity of community environments where Indigenous children live, the sample is not necessarily representative of the total population of Indigenous children in Australia [46]; accordingly, the results of this study may not be generalizable to this broader population of Indigenous children.

## Conclusion

Direct and persistent vicarious racial discrimination are detrimental to the physical and mental health of Indigenous children in Australia. Racism is thus a critical social determinant of health in Indigenous and minority children. Prolonged and more frequent exposure to racial discrimination that starts in the early lifecourse can impact on multiple domains of health in later life. Tackling and reducing racism should be an integral part of policy and intervention aimed at improving the health of Australian Indigenous children and thereby reducing health disparities between Indigenous and non-Indigenous children.

## Additional files

Additional file 1: Effect of imputation on odds ratio estimates and confidence intervals—mental health. (DOCX 26 kb)

Additional file 2: Effect of imputation on odds ratio estimates and confidence intervals—obesity. (DOCX 26 kb)

## Abbreviations

aOR: Adjusted odds ratio
BMI: Body mass index
CI: Confidence interval
CSEBD: Clinically significant emotional or behavioural difficulties
IRISEO: Index of Relative Indigenous Socioeconomic Outcomes
LORI: Level of Relative Isolation
LSIC: Longitudinal Study of Indigenous Children
OR: Odds ratio
PAR: Population attributable risk
SDQ: Strengths and Difficulties Questionnaire
SEIFA: Socioeconomic Index for Areas
SES: Socioeconomic status

## References

[1] Krieger N (2001). Theories for social epidemiology in the 21st century: an ecosocial perspective. Int J Epidemiol.

[2] Coll CG, Crnic K, Lamberty G, Wasik BH, Jenkins R, Garcia HV (1996). An integrative model for the study of developmental competencies in minority children. Child Dev.

[3] Myers HF (2009). Ethnicity-and socio-economic status-related stresses in context: an integrative review and conceptual model. J Behav Med.

[4] Zubrick SR, Shepherd CCJ, Dudgeon P, Gee G, Paradies Y, Scrine C, Walker R. Social determinants of social and emotional wellbeing. In: Dudgeon P, Walker R, Milroy H, editors. Working together: Aboriginal and Torres Strait Islander mental health and wellbeing principles and practice. Second edition Perth: Telethon Institute for Child Health Research, University of Western Australia, Commonwealth Government; 2014. p. 93-112.

[5] Paradies Y, Ben J, Denson N, Elias A, Priest N, Pieterse A (2015). Racism as a determinant of health: a systematic review and meta-analysis. PLoS One.

[6] Williams DR, Mohammed SA (2013). Racism and health I: pathways and scientific evidence. Am Behav Sci.

[7] Pascoe EA, Smart RL (2009). Perceived discrimination and health: a meta-analytic review. Psychol Bull.

[8] Paradies Y (2006). A systematic review of empirical research on self-reported racism and health. Int J Epidemiol.

[9] Schmitt MT, Branscombe NR, Postmes T, Garcia A (2014). The consequences of perceived discrimination for psychological well-being: a meta-analytic review. Psychol Bull.

[10] Lewis TT, Williams DR, Tamene M, Clark CR (2014). Self-reported experiences of discrimination and cardiovascular disease. Curr Cardiovasc Risk Rep.

[11] Williams DR, Priest N, Anderson NB (2016). Understanding associations among race, socioeconomic status, and health: patterns and prospects. Health Psychol.

[12] Lewis TT, Cogburn CD, Williams DR (2015). Self-reported experiences of discrimination and health: scientific advances, ongoing controversies, and emerging issues. Annu Rev Clin Psychol.

[13] Phelan JC, Link BG (2015). Is racism a fundamental cause of inequalities in health?. Annu Rev Sociol.

[14] Williams DR, Mohammed SA (2009). Discrimination and racial disparities in health: evidence and needed research. J Behav Med.

[15] Berman G, Paradies Y (2010). Racism, disadvantage and multiculturalism: towards effective anti-racist praxis. Ethnic Racial Stud.

[16] Paradies Y, Harris R, Anderson I (2008). The impact of racism on indigenous health in Australia and Aotearoa: towards a research agenda.

[17] Jones CP (2000). Levels of racism: a theoretic framework and a gardener’s tale. Am J Public Health.

[18] Williams DR, Whitfield KE (2004). Racism and health. Closing the gap: improving the health of minority elders in the new millennium.

[19] Williams DR, Neighbors HW, Jackson JS (2003). Racial/ethnic discrimination and health: findings from community studies. Am J Public Health.

[20] Brondolo E, Hausmann LR, Jhalani J, Pencille M, Atencio-Bacayon J, Kumar A (2011). Dimensions of perceived racism and self-reported health: examination of racial/ethnic differences and potential mediators. Ann Behav Med.

[21] Harrell SP (2000). A multidimensional conceptualization of racism-related stress: implications for the well-being of people of color. Am J Orthop.

[22] Priest N, Paradies Y, Trenerry B, Truong M, Karlsen S, Kelly Y (2013). A systematic review of studies examining the relationship between reported racism and health and wellbeing for children and young people. Soc Sci Med.

[23] Paradies Y (2016). Colonisation, racism and indigenous health. J Popul Res.

[24] Bécares L, Nazroo J, Kelly Y (2015). A longitudinal examination of maternal, family, and area-level experiences of racism on children’s socioemotional development: patterns and possible explanations. Soc Sci Med.

[25] Sanders-Phillips K (2009). Racial discrimination: a continuum of violence exposure for children of color. Clin Child Fam Psychol Rev.

[26] Pachter LM, Coll CG (2009). Racism and child health: a review of the literature and future directions. J Dev Behav Pediatr.

[27] Acevedo-Garcia D, Rosenfeld LE, Hardy E, McArdle N, Osypuk TL (2013). Future directions in research on institutional and interpersonal discrimination and children’s health. Am J Public Health.

[28] Mustillo S, Krieger N, Gunderson EP, Sidney S, McCreath H, Kiefe CI (2004). Self-reported experiences of racial discrimination and black-white differences in preterm and low-birthweight deliveries: the CARDIA study. Am J Public Health.

[29] Rankin KM, David RJ, Collins JW (2011). African American women’s exposure to interpersonal racial discrimination in public settings and preterm birth: the effect of coping behaviors. Ethn Dis.

[30] Rosenberg L, Palmer JR, Wise LA, Horton NJ, Corwin MJ (2002). Perceptions of racial discrimination and the risk of preterm birth. Epidemiology.

[31] Misra D, Strobino D, Trabert B (2010). Effects of social and psychosocial factors on risk of preterm birth in black women. Paediatr Perinat Epidemiol.

[32] Dominguez TP, Dunkel-Schetter C, Glynn LM, Hobel C, Sandman CA (2008). Racial differences in birth outcomes: the role of general, pregnancy, and racism stress. Health Psychol.

[33] Gee GC, Walsemann KM, Brondolo E (2012). A life course perspective on how racism may be related to health inequities. Am J Public Health.

[34] King M, Smith A, Gracey M (2009). Indigenous health part 2: the underlying causes of the health gap. Lancet.

[35] Priest N, Paradies Y, Stevens M, Bailie R (2012). Exploring relationships between racism, housing and child illness in remote indigenous communities. J Epidemiol Community Health.

[36] Zubrick SR, Silburn SR, Lawrence DM, Mitrou FG, Dalby RB, Blair EM (2005). The Western Australian Aboriginal child health survey: the social and emotional wellbeing of Aboriginal children and young people.

[37] Priest N, Paradies Y, Stewart P, Luke J (2011). Racism and health among urban Aboriginal young people. BMC Public Health.

[38] Priest NC, Paradies YC, Gunthorpe W, Cairney SJ, Sayers SM (2011). Racism as a determinant of social and emotional wellbeing for Aboriginal Australian youth. Med J Aust.

[39] Brody GH, Chen YF, Murry VM, Ge X, Simons RL, Gibbons FX (2006). Perceived discrimination and the adjustment of African American youths: a five-year longitudinal analysis with contextual moderation effects. Child Dev.

[40] Huynh VW, Gillen-O’Neel C (2013). Discrimination and sleep: the protective role of school belonging. Youth Soc.

[41] Bittencourt AA, Aerts DR, Alves GG, Palazzo L, Monteiro L, Vieira PC (2009). Feelings of discrimination among students: prevalence and associated factors. Rev Saude Publica.

[42] Kelly Y, Becares L, Nazroo J (2013). Associations between maternal experiences of racism and early child health and development: findings from the UK millennium cohort study. J Epidemiol Community Health.

[43] Shankardass K, McConnell R, Jerrett M, Milam J, Richardson J, Berhane K (2009). Parental stress increases the effect of traffic-related air pollution on childhood asthma incidence. Proc Natl Acad Sci.

[44] Northridge ME (1995). Public health methods--attributable risk as a link between causality and public health action. Am J Public Health.

[45] Thurber KA, Banks E, Banwell C (2015). Cohort profile: footprints in time, the Australian longitudinal study of indigenous children. Int J Epidemiol.

[46] Dodson M, Hunter B, McKay M (2012). Footprints in time: the longitudinal study of indigenous children: a guide for the uninitiated. Family Matters.

[47] Bennetts-Kneebone L, Christelow J, Neuendorf A, Skelton F (2012). Footprints in time: the longitudinal study of indigenous children: an overview. Family Matters.

[48] Farrant BM, Shepherd CCJ, Walker RD, Pearson GC. Early vocabulary development of Australian indigenous children: identifying strengths. Child Dev Res. 2014. doi:10.1155/2014/942817.

[49] Goodman R (1999). The extended version of the strengths and difficulties Questionnaire as a guide to child psychiatric Caseness and consequent burden. J Child Psychol Psychiatry.

[50] Goodman R, Ford T, Simmons H, Gatward R, Meltzer H (2000). Using the strengths and difficulties Questionnaire (SDQ) to screen for child psychiatric disorders in a community sample. Br J Psychiatry.

[51] Thurber KA (2012). Analyses of anthropometric data in the longitudinal study of indigenous children and methodological implications. Masters thesis. The Australian National University.

[52] Zubrick SR, Lawrence DM, Silburn SR, Blair E, Milroy H, Wilkes T (2004). Western Australian Aboriginal child health survey: the health of Aboriginal children and young people.

[53] Hunter B, Kennedy S, Smith D (2003). Household composition, equivalence scales and the reliability of income distributions: some evidence for indigenous and other Australians. Econ Rec.

[54] Hewitt B (2012). The longitudinal study of indigenous children: implications of the study design for analysis and results. LSIC technical report.

[55] Hildebrandt M, Bender R, Gehrmann U, Blettner M (2006). Calculating confidence intervals for impact numbers. BMC Med Res Methodol.

[56] Buuren S, Groothuis-Oudshoorn K (2011). Mice: multivariate imputation by chained equations in R. J Stat Softw.

[57] R: A language and environment for statistical computing. https://www.R-project.org/.

[58] McEwen BS (1998). Protective and damaging effects of stress mediators. N Engl J Med.

[59] Keating DP, Hertzman C (1999). Developmental health and the wealth of nations: social, biological, and educational dynamics.

[60] Coker TR, Elliott MN, Kanouse DE, Grunbaum JA, Schwebel DC, Gilliland MJ (2009). Perceived racial/ethnic discrimination among fifth-grade students and its association with mental health. Am J Public Health.

[61] Dahl RE, Lewin DS (2002). Pathways to adolescent health sleep regulation and behavior. J Adolesc Health.

[62] Kripke DF, Garfinkel L, Wingard DL, Klauber MR, Marler MR (2002). Mortality associated with sleep duration and insomnia. Arch Gen Psychiatry.

[63] Milam J, McConnell R, Yao L, Berhane K, Jerrett M, Richardson J (2008). Parental stress and childhood wheeze in a prospective cohort study. J Asthma.

[64] Sandberg S, Paton JY, Ahola S, McCann DC, McGuinness D, Hillary CR (2000). The role of acute and chronic stress in asthma attacks in children. Lancet.

[65] Department of Families, Housing, Community Services and Indigenous Affairs. Footprints in Time: The Longitudinal Study of Indigenous Children—Key Summary Report from Wave 1. FaHCSIA. 2009. https://www.dss.gov.au/national-centre-for-longitudinal-data/footprints-in-time-the-longitudinal-study-of-indigenous-children-lsic/key-summary-report-from-wave-1-2009. Accessed 15 Jan 2017.

