# Supplementary material for: The impact of racial discrimination on the health of Australian Indigenous children aged 5–10 years: analysis of national longitudinal data
Source: Int J Equity Health. 2017 Jul 3;16:116. doi: 10.1186/s12939-017-0612-0 (PMC5496226; doi:10.1186/s12939-017-0612-0)
Supplement: Supplementary file 1 — Effect of imputation on odds ratio estimates and confidence intervals—mental health. (DOCX 26 kb) [file 12939_2017_612_MOESM1_ESM.docx]

**Overview**

Supplementary Tables 1 and 2 present the results from multiple imputation alongside those of the models using complete cases only, for two outcomes—mental health and obesity, respectively. These were selected as mental health required no imputation of the outcome, only the independent variables, and obesity required imputation of both the outcome and independent variables. The consistency of results between the complete case data and the results using imputation means imputation was not pursued and the data with fewer assumptions (the complete case data) were presented as the main results.

**Supplementary Table 1. Effect of imputation on odds ratio estimates and confidence intervals—mental health**

|  | Mental health  Model 1^a^ (OR (95% CI)) | |  | Mental health  Model 2^b^ (OR (95% CI)) | |
| --- | --- | --- | --- | --- | --- |
|  | Complete cases^c^ | With imputation^d^ |  | Complete cases^c^ | With imputation^d^ |
| N | 789 | 1239 |  | 779 | 1239 |
| Primary carer’s experience of racism^e^ |  |  |  |  |  |
| Time-limited | 0.84 (0.56, 1.26) | 0.85 (0.59, 1.25) |  | 0.78 (0.51, 1.19) | 0.78 (0.53, 1.15) |
| Persistent | 1.68 (1.02, 2.74) | 1.63 (1.04, 2.54) |  | 1.46 (0.88, 2.45) | 1.47 (0.93, 2.30) |
| Family experience of racism, discrimination or prejudice^e^ |  |  |  |  |  |
| Time-limited | 1.00 (0.69, 1.45) | 1.02 (0.73, 1.42) |  | 0.95 (0.64, 1.39) | 0.98 (0.68, 1.41) |
| Persistent | 1.16 (0.76, 1.78) | 1.16 (0.77, 1.75) |  | 1.12 (0.71, 1.76) | 1.08 (0.72, 1.62) |
| Study child treated badly or discriminated against because  they were Aboriginal (ever) | 2.21 (1.48, 3.30) | 2.24 (1.50, 3.35) |  | 2.32 (1.52, 3.53) | 2.28 (1.51, 3.46) |

^a^ Model 1 is adjusted for sex and age of the study child.

^b^ Model 2 includes Model 1 variables as well as the age of the primary carer, level of relative isolation, primary carer education, and family financial strain.

^c^ Models exclude records with missing data.

^d^ Model estimates pooled from 10 imputed datasets, imputed via Multivariate Imputation by Chained Equations.

^e^ ‘Time-limited’ is defined as exposure to racism in one wave only, while ‘Persistent’ includes exposure in multiple waves.
